# A Novel 4H-SiC Asymmetric MOSFET with Step Trench

**DOI:** 10.3390/mi15060724

**Published:** 2024-05-30

**Authors:** Zhong Lan, Yangjie Ou, Xiarong Hu, Dong Liu

**Affiliations:** 1 School of Electrical Engineering, Southwest Jiaotong University, Chengdu 611756, China; lanzhong@my.swjtu.edu.cn (Z.L.); oyj194116@my.swjtu.edu.cn (Y.O.); 2 School of Science, Xihua University, Chengdu 610039, China; hxr2013@mail.xhu.edu.cn

**Keywords:** SiC MOSFET, step trench, figure of merit (FOM), crosstalk

## Abstract

In this article, a silicon carbide (SiC) asymmetric MOSFET with a step trench (AST-MOS) is proposed and investigated. The AST-MOS features a step trench with an extra electron current path on one side, thereby increasing the channel density of the device. A thick oxide layer is also employed at the bottom of the step trench, which is used as a new voltage-withstanding region. Furthermore, the ratio of the gate-to-drain capacitance (*C*_gd_) to the gate-to-source capacitance (*C*_gs_) is significantly reduced in the AST-MOS. As a result, the AST-MOS compared with the double-trench MOSFET (DT-MOS) and deep double-trench MOSFET (DDT-MOS), is demonstrated to have an increase of 200 V and 50 V in the breakdown voltage (BV), decreases of 21.8% and 10% in the specific on-resistance (*R*_on,sp_), a reduction of about 1 V in the induced crosstalk voltage, and lower switching loss. Additionally, the trade-off between the resistance of the JFET region (*R*_JFET_) and the electric field in the gate oxide (*E*_ox_) is studied for a step trench and a deep trench. The improved performances suggest that a step trench is a competitive option in advanced device design.

## 1. Introduction

Silicon carbide (SiC) has become one of the most attractive wide-bandgap-power semiconductor materials in recent years due to its outstanding properties, such as high breakdown field, high electron mobility, and high thermal conductivity [1,2,3,4]. As a unipolar representative device, an SiC metal–oxide–semiconductor field-effect transistor (MOSFET) can fully exploit the material superiority of SiC to achieve a lower specific on-resistance (*R*_on,sp_) and a higher switching frequency. This has been widely used in applications with high power density and efficiency, such as photovoltaic and new energy vehicles [5,6]. However, SiC MOSFET typically exhibits low channel mobility, a high electric field in the gate oxide (*E*_ox_), and a large threshold voltage shift, affecting the expected performance and long-term reliability of the operation of the device [7,8,9].

To address the issue of low channel mobility, it is acknowledged that the trench MOSFET (UMOSFET) is able to effectively increase channel density and improve channel mobility [10]. However, if the conventional trench structure is used in SiC UMOSFET, this leads to a high *E*_ox_. For this reason, with the SiC UMOSFET, it is necessary to introduce extra p-shield regions into a structure. In recent years, a number of novel shielding structures have been proposed for application to SiC UMOSFET to solve the problem of high *E*_ox_ [11,12,13,14,15]. Unfortunately, all these structures inevitably introduce additional resistance in the JFET region (*R*_JFET_). Lowering the *E*_ox_ while retaining superior forward characteristics remains a significant challenge in the development of high-performance SiC UMOSFET. The ROHM’s 3rd-generation double-trench MOSFET (DT-MOS) was the first commercially available SiC UMOSFET solution [16]. It introduces p-shield regions under the recessed source contact to lower the field near the gate bottom. However, it has recently been shown that the p-shield regions of the DT-MOS inadequately protect the gate oxide under a range of extreme operational conditions [17]. Therefore, in the ROHM’s 4th-generation deep double-trench MOSFET (DDT-MOS), the deeper p-shield regions and source trench are employed to further reduce the *E*_ox_ [18]. Nevertheless, although the DDT-MOS achieves a lower *E*_ox_, the source trench in the DDT-MOS still occupies a considerable cell area. This is not favorable to improving the channel density. One potential solution to this problem is to utilize the deep source trench as a gate trench, with one side of the trench acting as a channel and the other side being grounded to the source. However, since a p-pillar2 with a low doping concentration improves the breakdown voltage (BV) and a p-pillar1 with a high doping concentration reduces *E*_ox_ in the DDT-MOS, it is difficult to realize the asymmetric impurity distribution in the DDT-MOS via a single-step ion implantation process to ensure a low *E*_ox_ and a high BV of the device.

In this article, a SiC asymmetric MOSFET with step trench (AST-MOS) is proposed and studied via TCAD simulation. The AST-MOS features a step trench, with an additional electron current path on one side to increase the channel density of the device. A thick oxide layer is also applied at the bottom of the step trench. This is used as a new voltage-withstanding region. The two p-pillars with varying widths and concentrations are formed separately, which means that they do not affect each other. This makes it easier to realize an asymmetric impurity distribution without deteriorating the performance of device. Furthermore, the ratio of the gate-to-drain capacitance (*C*_gd_) to the gate-to-source capacitance (*C*_gs_) in the AST-MOS is significantly reduced. As a result, the AST-MOS exhibits better performance, including higher BV, lower specific on-resistance (*R*_on,sp_), reduced induced crosstalk voltage, and diminished switching loss, while maintaining a low *E*_ox_.

## 2. Device Structure and Mechanism

**Figure 1:**
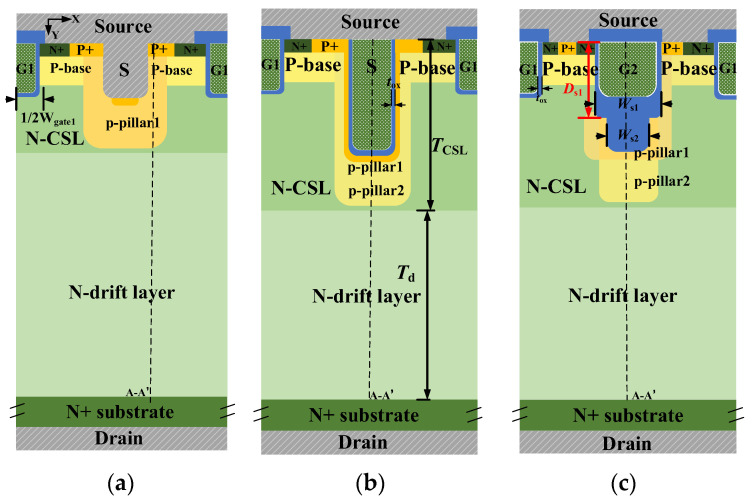
Schematic cross-sectional structures of the (**a**) DT-MOS, (**b**) DDT-MOS and (**c**) AST-MOS.

Figure 1 shows the schematic cross-sectional structures of the DT-MOS, DDT-MOS, and AST-MOS. The main structure parameters of the three structures are shown in Table 1. The three SiC UMOSFETs under study are rated for 1.2 kV, with a cell pitch of 2.6 μm and an active area of 10 mm^2^ for a fair comparison. The DT-MOS is shown in Figure 1a. For the DT-MOS, due to the simultaneous etching of the source and gate trench, the p-shield is formed at the bottom of the source trench through high-dose p-type ion implantation to ensure adequate shielding effects for the gate oxide. The DDT-MOS is shown in Figure 1b and is derived from the DT-MOS structure. However, the former has a deeper p-shield than the latter as a result of the deeper source trench. This allows for a greater level of protection to be afforded to the gate oxide. The AST-MOS is shown in Figure 1c. Compared with the DT-MOS and DDT-MOS, the AST-MOS transforms a deep trench into a step trench and employs a two-step ion implantation to form two p-pillars with varying widths and concentrations. The two p-pillars are formed separately, which means that they do not affect each other. The width of each p-pillar is greater than the width of each trench by 0.2 μm. To achieve the asymmetric structure, the p-pillar1 on the one side is compensated by injecting N-type impurities at an angle without affecting the other side p-pillar1. This increases channel density by 50%. The polysilicon in the step trench is connected to the gate and is isolated from the source metal. Accordingly, the AST-MOS may be regarded as a hybrid of the ROHM’s DT-MOS and the INFINEON’s asymmetric trench MOSFET (AT-MOS). Additionally, the AST-MOS features a thick oxide layer at the bottom of the step trench, which is used as a new voltage-withstanding region.

**Figure 2:**
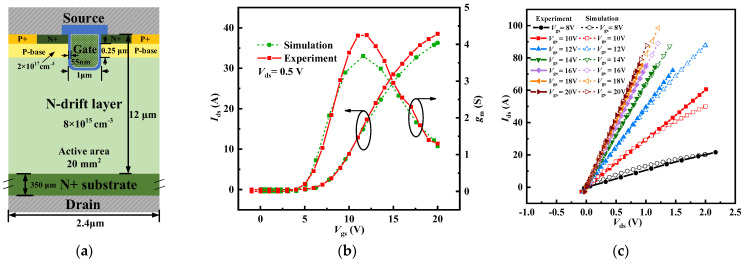
(**a**) Schematic cross-sectional structure and detailed information of the experimental device. Comparison of the experimental results and the simulation results: (**b**) *I*_ds_−*V*_gs_ and g_m_−*V*_gs_ curves at *V*_ds_ = 0.5 V, (**c**) *V*_ds_−*I*_ds_ curves with varying *V*_gs_.

In this study, Sentaurus TCAD 2018 tools are used to perform static and dynamic simulations of the device. A few crucial models are considered during the simulation, including Shockley–Read–Hall recombination, Okuto–Crowell impact ionization, Auger recombination, doping-dependent transport, enormal, bandgap narrowing, incomplete dopant ionization, and material anisotropy. The parameters of the TCAD simulation models are also calibrated via some experiments. Figure 2a shows the schematic cross-sectional structure and gives detailed information about the experimental device. The device is an unshielded trench MOSFET with a cell pitch of 2.4 μm and an active area of 20 mm^2^. Figure 2a,b show the comparison of the experimental results and the simulation results: the drain-source current (*I*_ds_) versus the gate-source voltage (*V*_gs_) curve and transconductance (g_m_) versus *V*_gs_ curve at a drain-source voltage (*V*_ds_) of 0.5 V, and *I*_ds_−*V*_ds_ curves with varying *V*_gs_. There is a high degree of overlap between the two, indicating that the subsequent simulation results in this paper are quite reliable. In the process of model calibration, the density of electron defects at different energy levels is modified and this is coupled with the appropriate bulk mobility to fit the *I*_ds_−*V*_gs_, g_m_−*V*_gs_, and *I*_ds_−*V*_ds_ curves. Threshold voltage is mainly fitted by adjusting the fixed charge density. When the thickness of the gate oxide is around 55 nm and the doping concentration of the P-base is 2 × 10^17^ cm^−3^, a fixed charge of 1.4 × 10^12^ cm^−2^ is employed and the measured threshold voltage is close to the simulated threshold voltage, both of which are approximately 4 V. For the hole defects, since they do not significantly affect the subsequent simulation results, this paper sets their density to be consistent with that of the electron defects and their energy level to be symmetrical to that of the electron defects.

**Figure 3:**
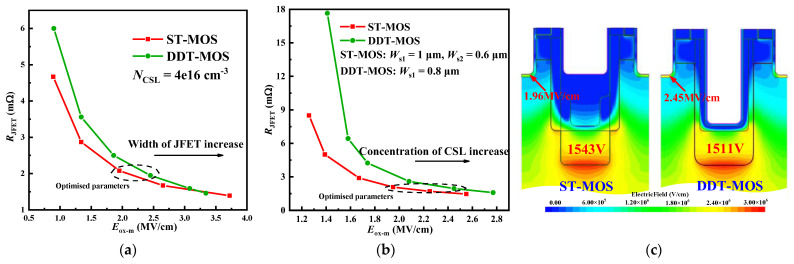
Trade-off between the *R*_JFET_ and the *E*_ox-m_ with varying (**a**) the width of JFET and (**b**) the concentration of CSL for the DDT-MOS and ST-MOS. (**c**) Electric field distribution of the DDT-MOS and ST-MOS at a maximum electric field in the SiC (*E*_sic-m_) of 3 MV/cm.

The *R*_JFET_ and the maximum electric field in the gate oxide (*E*_ox-m_) are often influenced by the p-shield. The significant disparity between the BV of the DT-MOS and that of the other two structures as well as the asymmetric design cause a higher *E*_ox_. In this paper, only the trade-off between the *E*_ox-m_ (*V*_ds_ = 1200 V, *V*_gs_ = 0 V) and *R*_JFET_ at *I*_ds_ = 40 A for the DDT-MOS and the SiC step trench UMOSFET (ST-MOS, parameters are the same as the AST-MOS) is investigated. Figure 3a and Figure 3b demonstrate the *R*_JFET_ versus the *E*_ox-m_ curves when varying the width of JFET and the concentration of current spreading layer (CSL) for the DDT-MOS and ST-MOS, respectively. With a larger width of JFET or a higher concentration of CSL, the *R*_JFET_ in the two structures is reduced owing to the less severe JFET effect induced by the p-shield, whereas the *E*_ox-m_ located at the trench corner is increased. The *R*_JFET_ versus the *E*_ox-m_ curve of the ST-MOS is more closely aligned with the zero-point compared with that of the DDT-MOS. This indicates that the step trench structure has a superior trade-off between the *R*_JFET_ and the *E*_ox-m_. Figure 3c provides an explanation for this. The ST-MOS employs multistage p-pillars, introducing multiple electric field peaks in the bulk of SiC to fully terminate the electric field lines in the p-pillar and forming multi-width JFET regions to optimise the distribution of *R*_JFET_. In other words, the ST-MOS allows a smaller JFET width to achieve a small *E*_ox-m_ while maintaining the same JFET resistance as the DDT-MOS.

**Figure 4:**
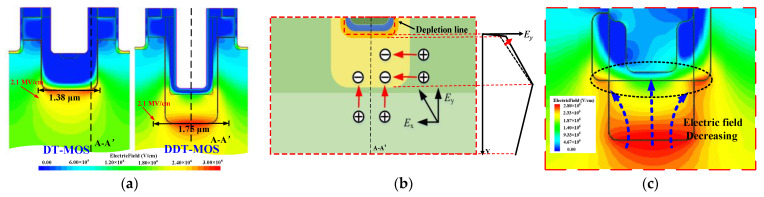
(**a**) Comparison of electric field distribution between the DT-MOS and the DDT-MOS during the off-state. (**b**) Analysis of the impact of the *E*_x_ on the *E*_y_ within the p-pillar2. (**c**) Effect of the thick oxide layer and the multistage p-pillar on electric field distribution.

In conventional SiC trench MOSFET, the p-shield is often designed with a high concentration in order to provide adequate protection for the gate oxide. In the DDT-MOS, the improved shielding effect allows for the use of a low concentration of the p-shield, which effectively improves the breakdown voltage (BV). Figure 4a,b demonstrate the physical mechanisms behind this. Firstly, when the p-pillar2 has a low concentration, it can effectively alleviate the concentration of the electric field at the corner of the p-pillar2. The high-electric-field region of the DDT-MOS is wider than that of the DT-MOS during the off-state. Furthermore, the PN junction relationship between the p-pillar2 and the N-epitaxial layer tends to be more like the parallel planar junction, which decreases the rate of change of the electric field in the vicinity of the junction, as shown in Figure 4a. Secondly, the p-pillar2 can also withstand part of the blocking voltage because of the depletion of the p-pillar2 by the transverse electric field (*E*_x_) and the longitudinal electric field (*E*_y_) during the off-state. As a consequence of the influence of the *E*_x_, the descent rate of the electric field is capable of being reduced for the longitudinal p-pillar2, as illustrated in Figure 4b. These two aspects result in a larger area being enclosed by the longitudinal electric field under electric fields of the same peak; namely, the device achieves a higher BV. Nevertheless, it must be noted that the second aspect has a lesser effect on the BV because the incomplete charge balance between the p-pillar2 and the CSL and the p-pillar2 is shallow in depth. For the AST-MOS, as illustrated in Figure 4c, the thick oxide layer within the step trench and the multistage p-pillar also has an effect on the BV. On the one hand, owing to the higher doping concentration of the p-pillar1, the electric field is concentrated at the p-pillar1, especially at the corner of the p-pillar1, which thus exhibits a wider multistage electric field distribution. On the other hand, the thick oxide layer is used for a new voltage-withstanding region, which also reduces the electric field near the PN junction.

**Figure 5:**
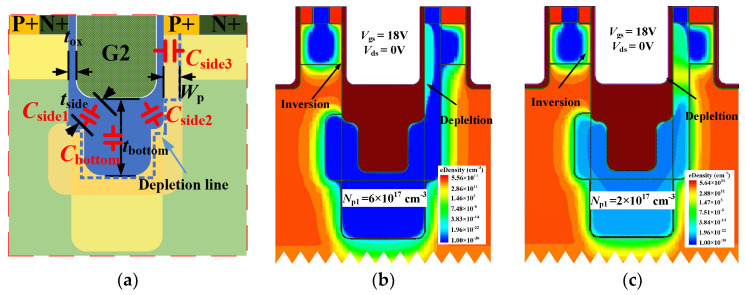
(**a**) Components of gate-to-pillar capacitance. Electron density distribution when (**b**) *N*_p1_ = 6 × 10^17^ cm^−3^ and (**c**) *N*_p1_ = 2 × 10^17^ cm^−3^ at *V*_gs_ = 18 V and *V*_ds_ = 0 V.

Since AST-MOS introduces an additional gate region, the *C*_gs_ of AST-MOS becomes larger compared to that of the other two structures. As shown in Figure 5a, the added capacitance, gate-to-pillar capacitance (*C*_gp_), is composed of four components of the AST-MOS, and can be expressed by Equation (1):

(1) $$C_{\mathrm{gp}}=C_{\mathrm{bottom}}+C_{\mathrm{side}1}+C_{\mathrm{side}2}+C_{\mathrm{side}3}$$

where the capacitance of the vertical bottom (*C*_bottom_) and the capacitance of the bottom side (*C*_side1_, *C*_side2_) are equivalent to the specific capacitance of the bottom and side oxide because the oxide layer at the bottom of the G2 is very thick.

For the the capacitance of the G2 right side (*C*_side3_), when a positive voltage is applied to the gate, there is no additional electron replenishment at the gate–p-pillar interface, and the p-pillar will be depleted without inversion, as shown in Figure 5b,c. The *C*_side3_ can be expressed as follows:

(2) $$C_{\mathrm{side}3}=\frac{C_{\mathrm{ox}-\mathrm{side}3}C_{\mathrm{sp}}}{C_{\mathrm{ox}-\mathrm{side}3}+C_{\mathrm{sp}}}$$

where *C*_ox-side3_ is the specific capacitance of the right gate oxide and *C*_sp_ is the depletion layer capacitance of the p-pillar.

## 3. Simulation Results and Analysis

**Figure 6:**
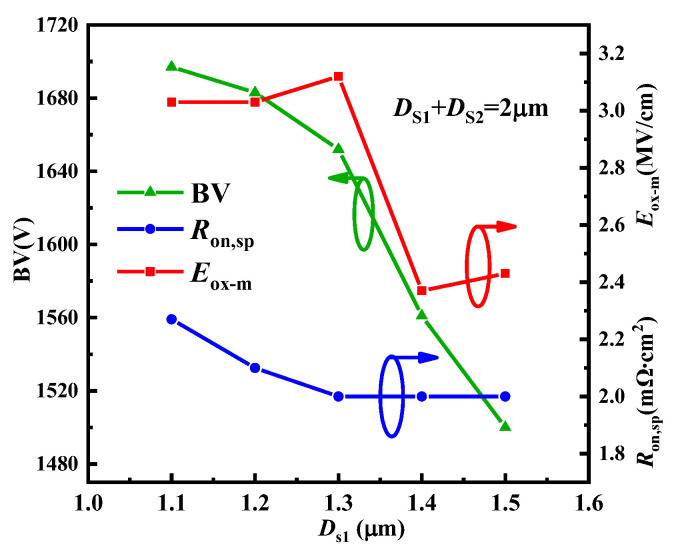
The influence of the *D*_s1_ on the BV, *E*_ox-m_ and *R*_on,sp_ when *D*_s2_ + *D*_s2_ = 2 μm.

In the AST-MOS, there are three key parameters, the depth of first step trench (*D*_s1_), the concentration of p-pillar1 (*N*_p1_), and the concentration of p-pillar2 (*N*_p2_), that need to be further optimized to obtain an excellent trade-off. Figure 6 shows the influence of the *D*_s1_ on the BV, the *E*_ox-m_, and the *R*_on,sp_ of the AST-MOS when *D*_s2_ + *D*_s2_ = 2 μm. It is indicated that the *R*_on,sp_ is sensitive to the *D*_s1_. This is mainly because the space between P-base and p-pillar1 mainly depends on *D*_s1_. If *D*_s1_ ≤ 1.3 μm, the space is narrow, causing a significant JFET effect between the P-base and p-pillar1. This means that the added current path has no effect due to its excessive on-resistance below the extra channel. When *D*_s1_ ≥ 1.3 μm, the on-resistance below the extra channel tends to stabilize, while the effective depth of the p-pillar2 decreases, resulting in a rapid decrease in the BV. Although the BV is greater when *D*_s1_ = 1.3 μm than when *D*_s1_ = 1.4 μm, the *E*_ox-m_ exceeds 3 MV/cm when *D*_s1_ = 1.3 μm. To achieve a smaller *R*_on,sp_, a higher BV, and a lower *E*_ox-m_, the *D*_s1_ is set to 1.4 μm. Furthermore, from the perspective of process realisation, a deep *D*_s1_ combined with a shallow *D*_s2_ is also a more reasonable approach. At this time, the *D*_s2_ is set to 0.6 μm. 

**Figure 7:**
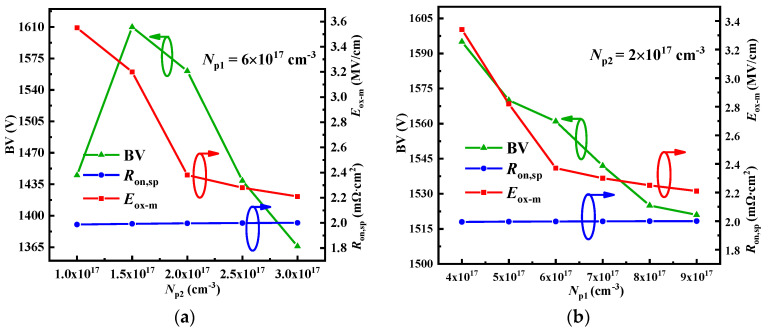
The influence of (**a**) the *N*_p2_ and (**b**) the *N*_p1_ on the BV, *E*_ox-m_ and *R*_on,sp_.

**Figure 8:**
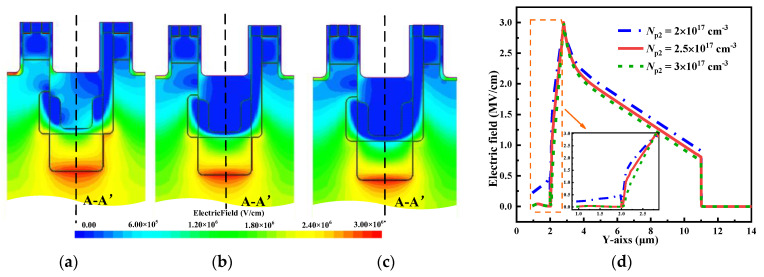
Electric field distribution when (**a**) *N*_p2_ = 2 × 10^17^ cm^−3^, (**b**) *N*_p2_ = 2.5 × 10^17^ cm^−3^ and (**c**) *N*_p2_ = 3 × 10^17^ cm^−3^. (**d**) Comparison of electric field distribution of p-pillar along with line A-A’ in Figure 1 with the different *N*_p2_ during the off-state.

Figure 7a exhibits the influence of the *N*_p2_ on the BV, *E*_ox-m_ and *R*_on,sp_ when *N*_p1_ = 6 × 10^17^ cm^−3^. It is evident that the *N*_p2_ has a negligible impact on the *R*_on,sp_. This is due to the high doping concentration of the CSL, which results in a small difference in *R*_JFET_. However, the influence on the BV and the *E*_ox-m_ of the device is significant. The reason for the influence on the *E*_ox-m_ is that the *N*_p2_ determines the number of electric field lines pointing towards the p-pillar2 during the off-state. A higher *N*_p2_ results in more electric field lines pointing towards the p-pillar2, resulting in an smaller electric field in the gate oxide. The influence on the BV is mainly due to the electric field distribution within the low-doped p-pillar2, which mainly depends on the *N*_p2_. A high *N*_p2_ causes the electric field to concentrate at the junction of the p-pillar2 and induces a narrower electric field distribution, as shown in Figure 8. Nevertheless, an excessively low *N*_p2_ can cause the electric field to concentrate on the p-pillar1 and the oxide layer, which may lead to a higher *E*_ox-m_ and a lower BV, as observed when *N*_p1_ = 1 × 10^17^ cm^−3^. It should be noted that when *N*_p2_ = 1.5 × 10^17^ cm^−3^, the BV is higher than when *N*_p2_ = 2 × 10^17^ cm^−3^. However, the *E*_ox-m_ is greater than 3 MV/cm; therefore, the *N*_p2_ is set to 2 × 10^17^ cm^−3^.

Figure 7b illustrates the influence of the *N*_p1_ on the BV, *E*_ox-m_, and *R*_on,sp_ when *N*_p2_ = 2 × 10^17^ cm^−3^. The *N*_p1_ also has a minimal effect on the *R*_on,sp_, the reason for which is the same as that behind the effect of *N*_p2_. However, the *N*_p1_ has a partial effect on the BV. The reason for which is that the *N*_p1_ can affect the electric field distribution within the p-pillar1 and oxide layer. When the *N*_p1_ is low, it possible to achieve a higher BV because the thick oxide layer can withstand a higher voltage. However, the *E*_ox-m_ may be more than 3 MV/cm due to the weakening of the shielding effect. When the *N*_p1_ is high, the electric field is concentrated on the p-pillar1, leading to a premature breakdown of the device or a reduction in the withstanding voltage within the thick oxide layer. Analyzing *N*_p1_ = 5 × 10^17^ cm^−3^, it should be noted that the BV is slightly higher than when *N*_p2_ = 6 × 10^17^ cm^−3^ and the *E*_ox-m_ is lower than 3 MV/cm. However, the increase in the *E*_ox-m_ is greater, while the improvement in the BV is smaller, when the *N*_p1_ is 5 × 10^17^ cm^−3^ instead of 6 × 10^17^ cm^−3^. Therefore, the *N*_p1_ is set to 6 × 10^17^ cm^−3^.

**Figure 9:**
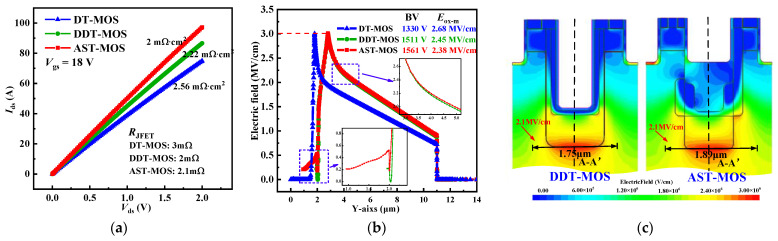
(**a**) On-state characteristics curve. (**b**) Electric field distribution of the AST-MOS, DT-MOS, and DT-MOS along line A-A’ at *E*_sic-m_ = 3 MV/cm. (**c**) Electric field distribution of the DDT-MOS and AST-MOS during the off-state.

Figure 9a shows the on-state characteristics curve of the three structures. It can be seen from Figure 9a that the *R*_on,sp_ values extracted at *V*_ds_ = 1 V are 2.56, 2.22, and 2.0 mΩ·cm^2^ for the DT-MOS, DDT-MOS, and AST-MOS, respectively. Owing to the high doping concentration of the JFET and drift region, and the extra electron current path. The AST-MOS achieves 21.8% and 10% reductions in *R*_on,sp_ compared with the DT-MOS and the DDT-MOS, respectively. The *R*_JFET_ values of the three structures are also shown in Figure 9a. The AST-MOS and the DDT-MOS employ a concentration of the CSL that is twice as great as that of the DT-MOS. Consequently, a lower *R*_JFET_ is also obtained, despite the depth of the JFET region of the two structures being deeper than that of the DT-MOS.

Figure 9b shows the electric field distribution of the three structures along line A-A’ at *E*_sic-m_ = 3 MV/cm. The BV extracted at *E*_sic-m_ = 3 MV/cm are 1330, 1511, and 1561 V for the DT-MOS, DDT-MOS, and AST-MOS, respectively. The *E*_ox-m_ values are 2.68, 2.45, and 2.38 MV/cm for the three structures, respectively. It is worth noting that the AST-MOS achieves 231 and 50 V increases in the BV compared with the DT-MOS and DDT-MOS, respectively, while maintaining the lowest *E*_ox-m_ value. This phenomenon is mainly attributed to the wider *W*_s1_ and the multistage p-pillar, which provide superior shielding effects even if there is no p-shield on one side of the first step trench. Further analysis of the BV enhancement can be attributed to two aspects, as shown in the small box in Figure 9b. On the one hand, part of the voltage is withstood by the thick oxide layer. On the other hand, the electric field decreases slightly slower across the PN junction due to the wider electric field distribution, as shown in Figure 9c. This is consistent with what is described in the mechanism section above. 

**Figure 10:**
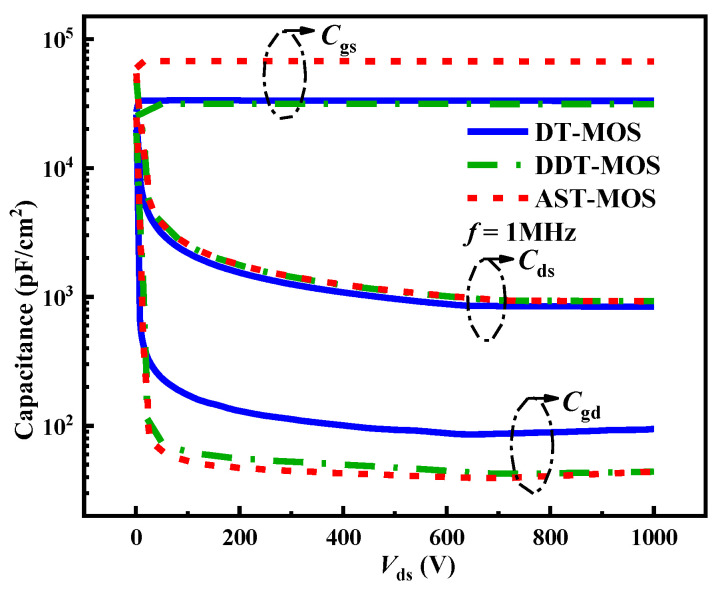
Capacitances of the DT-MOS, DDT-MOS and AST-MOS.

The capacitances of the three structures are shown in Figure 10. The gate-to-source capacitance (*C*_gd_) values extracted at *V*_ds_ = 1000 V are 94.5, 44, and 44 pF/cm^2^ for the DT-MOS, DDT-MOS, and AST-MOS, respectively. Although the AST-MOS has larger gate region numbers than the DT-MOS and DDT-MOS, the AST-MOS achieves a low *C*_gd_, the reasons for which are twofold. Primarily, the depth of the p-pillar is deeper than that of the DT-MOS, and the width of the p-pillar is wider than that of the DDT-MOS. These configurations are beneficial to the positive charge in the depletion layer overlapping gate and drain interacting with the p-pillar. Furthermore, as the p-shield of the AST-MOS overlaps with the gate more than that of the DT-MOS and DDT-MOS, the *C*_gs_ of the AST-MOS increases significantly. As shown in Figure 10, the gate-to-source capacitance (*C*_gs_) of the AST-MOS is 66.8 nF/cm^2^, while those of the DT-MOS and DDT-MOS are only 31.6 and 31.2 nF/cm^2^. The *C*_gs_ of the AST-MOS is more than 2 double that of the DT-MOS and DDT-MOS. Finally, the drain-source capacitance (*C*_ds_) is 850, 928, and 924 pF/cm^2^ for the DT-MOS, DDT-MOS, and AST-MOS, respectively.

**Figure 11:**
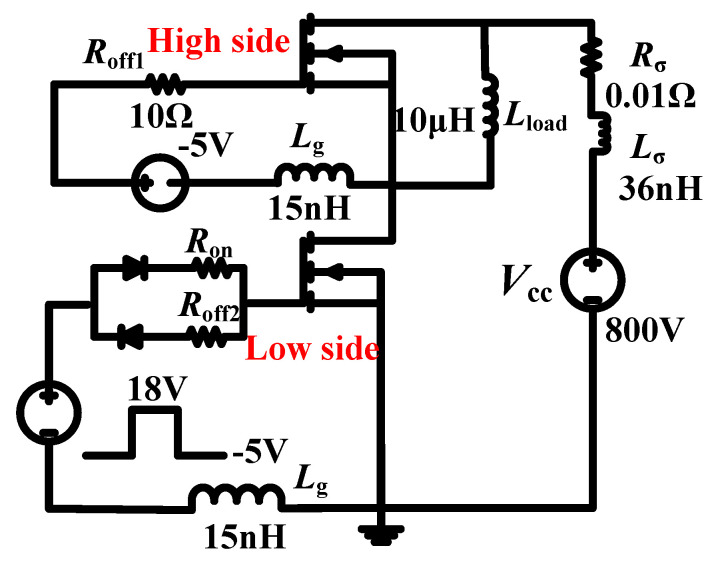
Switching-characteristics simulation circuit.

Since the AST-MOS has a large *C*_gs_ and a small *C*_gd_, the AST-MOS is able to achieve a small *C*_gd_/*C*_gs_, which is beneficial for the suppression of induced crosstalk voltage [19]. Currently, due to the problem of parasitic turn-on (PTO) [20], the switching frequency of the device is often limited by the gate-induced crosstalk voltage. The gate-induced crosstalk voltage mainly comes from two parts: one is the charging and discharging displacement current of the *C*_gd_, and the other is the induced voltage introduced by the common-source inductor (*L*_s_). Both of these are coupled to the gate voltage through the drive loop [21]. The two parts of the induced crosstalk voltage are often opposites [22]. Owing to the first part of these being unfavorable to the device, only the first part of these is considered in this paper in our efforts to establish a switching-characteristics simulation circuit, as shown in Figure 11. The circuit consists of high and low sides, where the high side is in a reverse conducting mode and the low side is in a switching mode. Working according to [23,24], gate inductance (*L*_g_) and parasitic inductance (*L*_δ_) are chosen to be 15 and 36 nH, respectively; the gate-to-source voltage (*V*_gs_) is a pulse voltage of +18 V/−5 V. The load inductance (*L*_load_) is set to 10 μH and the high-side gate turn-off resistance is 10 Ω. The *V*_gs_ of the high side during the switching process is given by Equation (3).

(3) $$V_{\mathrm{gs}}=V_{\mathrm{gsoff}}+R_{\mathrm{goff}}[C_{\mathrm{gd}}\frac{dV_{\mathrm{ds}}}{\mathrm{dt}}-(C_{\mathrm{gs}}+C_{\mathrm{gd}})\frac{dV_{\mathrm{gs}}}{\mathrm{dt}}]$$

where *V*_gsoff_ is the high side gate turn-off voltage, *R*_goff_ is the high side gate turn-off resistance, *V*_ds_ is the high side drain-to-source voltage, and *V*_gs_ is the high side gate-to-source voltage.

**Figure 12:**
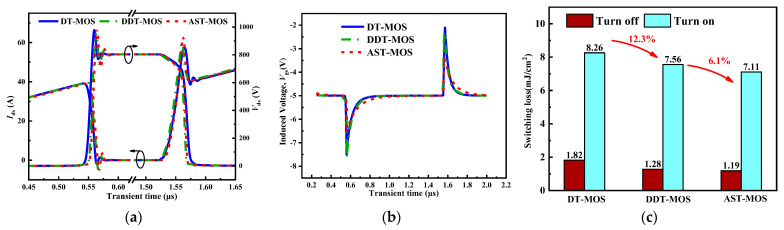
(**a**) Low-side switching waveform, (**b**) high-side gate-induced crosstalk waveform, and (**c**) low-side switching loss for AST-MOS, DDT-MOS, and DT-MOS.

When the gate turn-on resistance is equal to turn-off, the values are 10 Ω, 10 Ω, and 6 Ω for the DT-MOS, DDT-MOS, and AST-MOS, respectively. The d*I*_ds_/dt during the turn-on and turn-off processes for the three structures are similar, as shown in Figure 12a. The high-side gate-induced crosstalk voltage waveform for the three structures is shown in Figure 12b. The high-side positively induced crosstalk voltage (*V*_ic+_) is 2.9 V, 2.7 V, and 1.6 V for the DT-MOS, DDT-MOS, and AST-MOS, respectively, and the high-side negatively induced crosstalk voltage (*V*_ic−_) is −2.5 V, −2.6 V, and −1.6 V for the DT-MOS, DDT-MOS, and AST-MOS, respectively. The AST-MOS exhibited lower *V*_ic+_ values of 1.3 V and 1.1 V compared to that of the DT-MOS and DDT-MOS during the turn-on process. Similarly, during the turn-off process, the *V*_ic−_ of the AST-MOS is 0.9 V and 1 V lower than that of the DT-MOS and DDT-MOS. This indicates that a larger gate turn-off voltage can be employed for the AST-MOS during the switching process. Figure 12c illustrates the switching loss of the three structures. The AST-MOS exhibits a lower switching loss at a similar d*I*_ds_/dt. 

**Figure 13:**
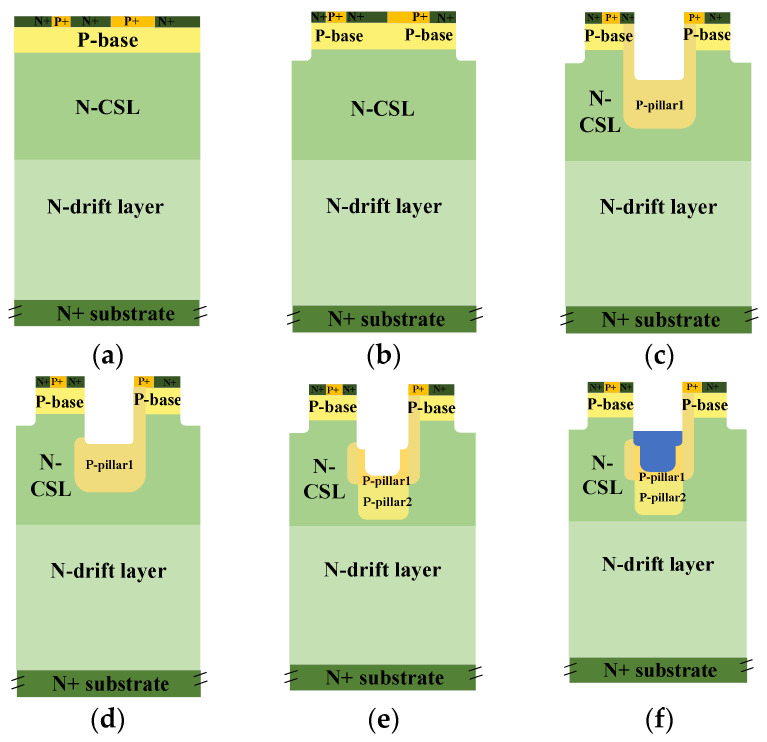

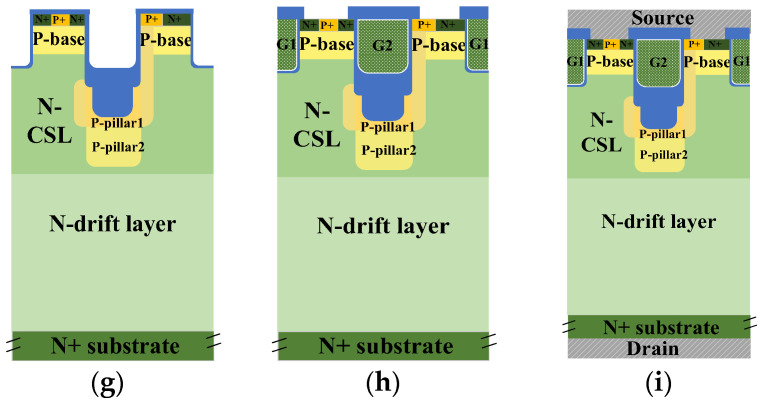
Process flow for the AST-MOS. (**a**) Epitaxial layer preparation and formation of the P-base, N+ source region, and P+ source region. (**b**) Etching the gate trench G1. (**c**) Etching the first step trench and forming the p-pillar1. (**d**) Forming an extra channel on one side of the deep trench. (**e**) Etching the second step trench and forming the p-pillar2. (**f**) Depositing oxide and back-etching. (**g**) Depositing gate oxide. (**h**) Depositing polysilicon and ILD, and then patterning. (**i**) Metallization.

Figure 13 shows the main process flow for the AST-MOS. The epitaxial wafer with the CSL is prepared; then, the N+ source region, P+ source region, and P-base are formed by ion implantation at 500 °C (see Figure 13a). The gate trench G1 is etched (see Figure 13b). The first step trench is etched and then the first p-pillar, with specified width and depth, is formed by tilted injection (see Figure 13c). The extra channel on one side of the deep trench is formed via tilted N-type ion implantation (see Figure 13d). The second step trench is etched and then the second p-pillar is formed via tilted injection (see Figure 13e). The thick oxide is deposited. Then, the oxide is back-etched to a specified depth and ion injection is performed at 1750 °C. Then, the silicon carbide surface is repaired via annealing in H_2_ atmosphere at 1400 °C (see Figure 13f). Gate oxide is deposited, followed by annealing in NO atmosphere (see Figure 13g). Polysilicon is deposited and back-etched to a specified depth, followed by the ILD deposition and patterning (see Figure 13h). N+ source region, P+ source region, and backside drain are sputtered with Ni to form an ohmic contact; this is followed by metal thickening and PI deposition and patterning (see Figure 13i). 

Table 2 shows the performance comparison of the three structures. Compared with the DT-MOS and the DDT-MOS, the AST-MOS features a thick oxide layer at the bottom step trench and a low-doped p-pillar2, resulting in increase of 231 V and 50 V increases in the BV, respectively, while maintaining a low *E*_ox-m_. Additionally, due to the high doping concentration of the JFET and drift regions and the extra electron current path, the AST-MOS achieves 21.8% and 10% reductions in the *R*_on,sp_, respectively. Meanwhile, the *C*_gd_/*C*_gs_ of the AST-MOS experiences a significant decrease, resulting in a reduction of about 1 V in the induced crosstalk voltage and a lower switching loss at a similar d*I*_ds_/dt.

## 4. Conclusions

A novel 4H-SiC asymmetric MOSFET with a step trench is proposed and investigated via numerical simulation. The introduction of the step trench optimizes compromise between the *R*_JFET_ and *E*_ox-m_ and the asymmetric design improves the channel density of the device. A thick oxide layer is also employed at the bottom of the step trench to be used for a new voltage-withstanding region. As a result, the AST-MOS exhibits a higher BV and a lower *R*_on,sp_ than the DT-MOS and DDT-MOS. Additionally, due to the ratio of the *C*_gd_/*C*_gs_ in the AST-MOS decreasing, the AST-MOS demonstrates lower induced crosstalk voltage and switching losses. Consequently, the proposed AST-MOS has the potential to be a highly effective solution for designing advanced device structure. 

## Figures and Tables

**Table 1:** Structural parameters of the three devices.

Symbol	Description	DT-MOS	DDT-MOS	AST-MOS
*W*_cell_	Width of cell pitch, μm	2.6	2.6	2.6
*t*_ox_	Thickness of gate oxide, nm	55	55	55
*t*_gate1_	Thickness of gate oxide, μm	1	1	1
*W*_gate1_	Width of gate trench, μm	0.6	0.6	0.6
*T*_sub_	Thickness of substrate, μm	200	200	200
*N*_sub_	Concentration of substrate, cm^−3^	2.8 × 10^18^	2.8 × 10^18^	2.8 × 10^18^
*T*_pbase_	Thickness of P-base, μm	0.5	0.5	0.5
*N*_pbase_	Concentration of P-base, cm^−3^	2 × 10^17^	2 × 10^17^	2 × 10^17^
*D*_s1_	Depth of first step trench, μm	1	2	1.4
*D*_s2_	Depth of second step trench, μm	-	-	0.6
*W*_s1_	Width of first step trench, μm	0.8	0.8	1
*W*_s2_	Width of second step trench, μm	-	-	0.6
*D*_p1_	Depth of p-pillar1, μm	0.8	0.1	0.7
*D*_p2_	Depth of p-pillar2, μm	-	0.8	0.8
*N*_p1_	Concentration of p-pillar1, cm^−3^	1 × 10^18^	2 × 10^18^	6 × 10^17^
*N*_p2_	Concentration of p-pillar2, cm^−3^	-	2 × 10^17^	2 × 10^17^
*T*_d_	Thickness of N-drift, μm	9.1	8.1	8.1
*N*_d_	Concentration of N-drift, cm^−3^	8 × 10^15^	8 × 10^15^	8 × 10^15^
*T*_csl_	Thickness of CSL, μm	1.9	2.9	2.9
*N*_csl_	Concentration of CSL, cm^−3^	2 × 10^16^	4 × 10^16^	4 × 10^16^

**Table 2:** Comparison of the three devices’ characteristics.

Symbol	DT-MOS	DDT-MOS	AST-MOS
BV [V]	1330	1511	1561
*E*_mox_ [MV/cm]	2.68	2.45	2.38
*R*_on,sp_ [mΩ·cm^2^]	2.56	2.22	2
BFOM ^a^ [MW/cm^2^]	519.5	680.6	780.5
*C*_gs_ [nF/cm^2^]	31.6	31.2	66.8
*C*_gd_ [pF/cm^2^]	94.5	44	44
*E*_on_ [mJ/cm^2^]	25.4	24.2	23.3
*E*_off_ [mJ/cm^2^]	6.8	5.2	5.1
*C*_gd_ × *R*_on,sp_ [pF·mΩ]	241.9	96.8	88
*V*_ic+_ [V]	2.9	2.7	1.6
*V*_ic−_ [V]	−2.5	−2.6	−1.6

^a^ BFOM is the value of BV^2^/*R*_on,sp_.

## Data Availability

Data are contained within the article.
